# Acute Pancreatitis as the Index Manifestation of Parathyroid Adenoma

**DOI:** 10.7759/cureus.16948

**Published:** 2021-08-06

**Authors:** Mahalingam Sudharshan, Ranjith Kumaran, Sudharsanan Sundaramurthi, Balamourougan Krishnaraj, Sarath Chandra Sistla

**Affiliations:** 1 Surgery, Jawaharlal Institute of Postgraduate Medical Education and Research, Puducherry, IND; 2 General Surgery, Jawaharlal Institute of Postgraduate Medical Education and Research, Puducherry, IND

**Keywords:** hyperparathyroidism, hypercalcemia, spect, intestinal obstruction, tc-99m sestamibi

## Abstract

Acute pancreatitis is one of the most common clinical emergencies encountered in our day-to-day practice. Although gallstones are the most common cause worldwide, alcohol consumption remains the leading cause of acute pancreatitis in the Indian population. We report a rare case of parathyroid adenoma, which presented with acute pancreatitis as its initial manifestation in an elderly patient. A 65-year-old gentleman with acute abdominal pain, distension, and obstipation, underwent emergency laparotomy in view of acute intestinal obstruction and was found to have acute pancreatitis intra-operatively. On post-operative evaluation, his serum calcium was >14 mg/dl and serum parathormone (PTH) was >2,000 pg/ml. Single-photon emission computed tomography (SPECT) and technetium (Tc-99m) sestamibi scintigraphy revealed a right inferior parathyroid adenoma, which was surgically excised, following which the patient made an uneventful recovery. Hypercalcemia induced by hyperparathyroidism causes auto-activation of pancreatic enzymes within the pancreatic parenchyma and is also believed to cause pancreatic duct obstruction by calcium deposition, thus causing pancreatitis. Radionucleotide scan, in addition to contrast-enhanced computed tomography, can help in localizing the lesion causing hyperparathyroidism. Appropriate resuscitation and stabilization with anti-hypercalcemic measures, including hydration and forced calciuresis, followed by surgery form the mainstay of treatment in patients with primary hyperparathyroidism. Patients with acute pancreatitis without a history of gallstone disease or alcohol intake should be evaluated for other rare causes. Early diagnosis and prompt treatment of the underlying condition can prevent the recurrence of pancreatitis.

## Introduction

Acute pancreatitis is an inflammatory condition of the pancreas and is one of the leading causes of hospital admissions worldwide. It can be a potentially fatal condition with an overall mortality of up to 20% [1]. The causes of acute pancreatitis are diverse, among which gallstones and alcoholism are the leading causes. Considering the hazards of delayed diagnosis of acute pancreatitis, awareness of infrequent causes and unusual presentation is of paramount importance. One such rare cause is hyperparathyroidism with hypercalcemia. This causative association between hypercalcemia and pancreatitis is suggested by the fact that parathyroidectomy prevents the recurrence of pancreatitis in patients with hyperparathyroidism [2]. Here we report a rare case of parathyroid adenoma, which presented with acute pancreatitis as its initial manifestation.

## Case presentation

A 65-year-old gentleman, with a known case of chronic liver disease, presented with complaints of diffuse abdominal distension for two days associated with non-radiating, intermittent, colicky peri-umbilical pain. He had multiple episodes of bilious vomiting. He had not passed stool for one week and flatus for three days. There were no similar events in the past. He was not a known tobacco or alcohol abuser. He underwent no previous surgery.

On examination, he was found to be dehydrated with tachycardia. His abdomen was grossly distended and tender with diffuse involuntary guarding and rigidity. There was no mass palpable in the abdomen, no free fluid, and the hernial orifices were free. The bowel sound was absent. Digital rectal examination revealed collapsed and empty rectum with normal stool staining. His other systemic examination findings were clinically unremarkable. 

**Figure 1:**
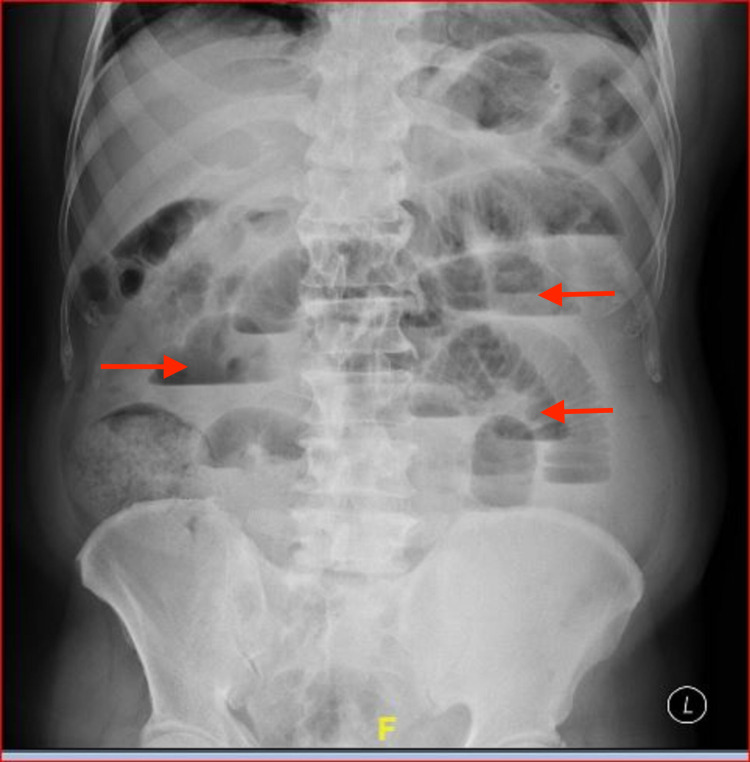
Plain radiograph of the abdomen - erect view showing multiple dilated small bowel loops with air-fluid levels (arrows).

His blood investigations revealed elevated blood urea nitrogen of 118 mg/dl and creatinine level of 3.6 mg/dl, suggestive of acute on chronic kidney disease, a normal hemogram, and a serum amylase of 207 IU/L. The serum amylase was at the upper limit of normal, and this was anticipated as such mild elevation is common in acute abdominal emergencies. A plain radiograph of the abdomen revealed multiple dilated small bowel loops with air-fluid levels, and ultrasonography (USG) of the abdomen revealed dilated aperistaltic small bowel loops (Figure 1).

**Figure 2:**
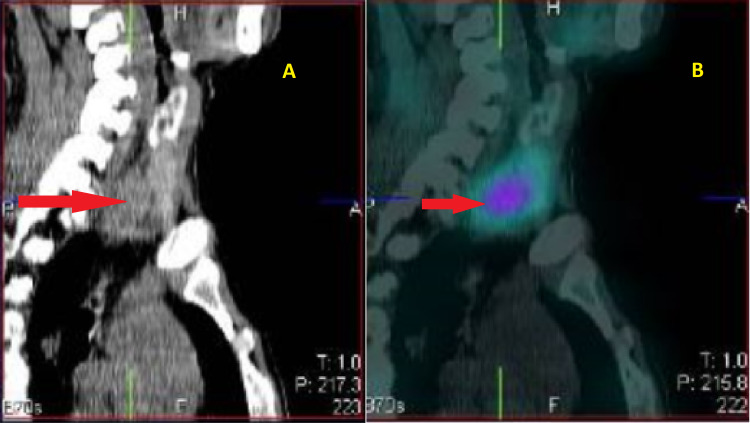
(A) Three-dimensional CT scan and (B) SPECT scintigraphy showing a right inferior parathyroid adenoma (arrow). SPECT, single-photon emission computed tomography.

**Figure 3:**
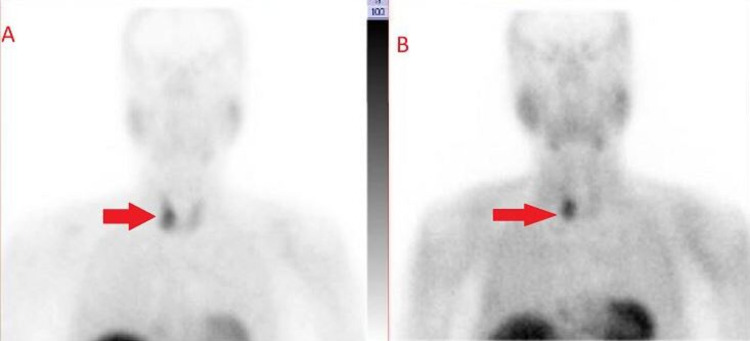
(A) Technetium (Tc-99m) sestamibi nucleotide scan showing increased tracer uptake in a soft tissue lesion in the right para-tracheal region. (B) Delayed images showed persistent tracer retention in the focal uptake suggestive of an adenoma in the right inferior parathyroid gland (arrow).

Thus, the diagnosis of acute intestinal obstruction was considered. A contrast-enhanced computed tomography (CECT) could not be done owing to a long waiting list for the procedure and also due to underlying renal failure in the patient. He was taken for emergency laparotomy as X-ray showed a classical picture of bowel obstruction and also for fear of poor outcomes in intestinal obstruction while waiting for CECT. As the patient was symptomatic for one week and with adhesive intestinal obstruction ruled out, as there were no prior abdominal surgeries in the past, conservative management was not considered. Intra-operatively, diffuse saponification of omental fat, inflamed lesser sac with extensive saponification, and calcification suggestive of acute pancreatitis were noted. The small and large bowel loops were grossly distended but viable with no obvious transition point. The dilated colonic loops were decompressed with a flatus tube. On further investigations to identify the cause of acute pancreatitis, he had hypercalcemia of 14 mg/dl. Thus, serum parathormone (PTH) levels were tested and found to be more than 2,000 pg/ml. USG of the neck was suggestive of a right inferior parathyroid adenoma. It was then confirmed by technetium (Tc-99m) sestamibi single-photon emission computed tomography (SPECT), which showed a focus of increased tracer uptake in a soft tissue nodule measuring 2.5 × 2.1 cm in the right para-tracheal region and posterior to the inferior pole of the right lobe of the thyroid. Delayed images showed persistent tracer retention in the focal uptake suggestive of an adenoma in the right inferior parathyroid gland (Figures 2, 3). Thus, he was diagnosed to have primary hyperparathyroidism (PHPT)-induced hypercalcemia causing acute pancreatitis.

Meanwhile, he underwent one session of hemodialysis along with anti-hypercalcemic measures, including intravenous fluids with forced calciuresis with diuretics to normalize his serum calcium levels. After proper stabilization and thorough pre-operative work-up, he underwent excision of the right inferior parathyroid adenoma under general anesthesia. The frozen section of the specimen was consistent with adenoma without any evidence of malignancy. His post-operative PTH level was 135.6 pg/ml, and calcium was 8.7 mg/dl. His post-operative period was uneventful, and he was thus discharged in a stable condition.

## Discussion

Pancreatitis is one of the most common surgical emergencies encountered. The worldwide incidence of acute pancreatitis ranges from 5 to 80 per 100,000 population [3]. Gallstone disease and alcoholism are the most frequent etiologies accounting for about 40% and 35% of the cases, respectively [4]. Though a majority of patients fall under these groups, emphasis should be made to identify other infrequent causes of pancreatitis in patients without a history of alcohol intake or gallstones. These include hypertriglyceridemia (1%-4%), drug-induced pancreatitis (2%-4.8%), trauma (1%), infections, and anatomical and genetic abnormalities. These are often missed at the initial evaluation, prolonging the hospital stay of patients and leading to a delay in administering the appropriate treatment. Hypercalcemia, with its associated factors, is one of these rare causes (<1%), which requires due consideration [5].

Hypercalcemia secondary to primary hyperparathyroidism (PHPT) is believed to play a significant role in acute pancreatitis. It is estimated that about 1.5% of patients with PHPT develop acute pancreatitis [6]. PHPT is an endocrine disorder where there is an endogenous excess production of parathyroid hormone. It is the third common endocrine disorder following diabetes mellitus and thyroid disorders and is the most common cause of hypercalcemia among non-hospitalized patients. The prevalence of acute pancreatitis in PHPT varies from 1.5% to 13% [7]. Such low prevalence can be explained by the occurrence of pancreatitis only at advanced stages of parathyroid disease. Nevertheless, on the contrary, in our patient, acute pancreatitis was the first clinical presentation. 

The most common cause of PHPT is parathyroid adenoma, which belongs to a spectrum of parathyroid proliferative disorders. Single gland adenoma is the most common pathology attributing to 80%-85% of cases, double adenomas in 4% of cases, multi-glandular hyperplasia in 10%-15% of cases, and rarely parathyroid carcinoma in less than 1% of cases [8]. 

Most of the patients with PHPT remain asymptomatic and are incidentally recognized by routine biochemical screening, showing elevated serum calcium and PTH levels. Thus, the classical presentation of PHPT is seldom seen. There may be non-specific symptoms such as fatigue, sleep and memory disturbances, depression, musculoskeletal pain, polyuria, and polydipsia. High levels of PTH cause bone demineralization and resorption leading to osteopenia, osteoporosis, and even cyst formation and fibrosis. PTH also mediates increased absorption of calcium from the gut and renal tubules, and the resultant hypercalcemia plays a pathognomic role in a majority of the clinical manifestations. This may include hypercalciuria (35%-40% cases) and nephrolithiasis (20% cases) as there is increased calcium load on the kidneys than its re-absorptive capacity [9]. The gastrointestinal manifestations include anorexia, nausea, vomiting, constipation, gastroesophageal reflux disease, and rarely acute pancreatitis. Our patient presented with a different clinical spectrum of acute pancreatitis with diffuse abdomen distension, colicky pain, and obstipation mimicking intestinal obstruction, probably due to ileus.

It is believed that hypercalcemia causes auto-activation of pancreatic enzymes like trypsinogen within the pancreatic parenchyma causing auto-digestion of the pancreas. It also causes the deposition of calcium in the pancreatic duct, causing its obstruction, which in turn triggers the enzyme activation [10]. Shah et al., in their study on 153 symptomatic PHPT patients, found that serum calcium level was associated with a 1.3 times higher risk of developing acute pancreatitis [11]. 

There exists a negative feedback mechanism exerted by serum calcium on PTH secretion. In addition to the elevated serum calcium and PTH levels, normal PTH in the setting of high calcium is an indication of PHPT, similarly high PTH in the presence of normal calcium level is called normocalcemic PHPT. Thus, the assessment of serum calcium levels combined with PTH levels helps in identifying hyperparathyroidism. 

Once PHPT is confirmed, the next step is to locate the abnormally secreting gland. USG of the neck is the initial imaging modality where parathyroid adenomas are typically homogeneously hypo-echoic when compared with the adjacent thyroid and can be detected with ease when they are larger than 1 cm, while normal glands are too small to be detected [12]. On the other hand, technetium (Tc-99m) labeled with sestamibi has a high affinity for mitochondria of parathyroid and thus has a prolonged and avid uptake in adenomas. Though it cannot differentiate benign from malignant lesions, it helps to identify the presence of ectopic parathyroid, local recurrence, or distant metastasis in cases of parathyroid carcinoma. 

Single-photon emission computed tomography (SPECT) has a high sensitivity of localizing enlarged parathyroid glands by scintigraphy, which facilitates three-dimensional identification of an enlarged parathyroid gland. Thus, combined SPECT and CT help in better localization of the lesion [12]. More recently, four-dimensional CT, which includes three-dimensional CT scanning with perfusion information derived from non-contrast, arterial, and delayed (venous) phase imaging, is being employed [13]. These imaging modalities provide precise anatomical and functional details of the gland and guide the surgeon for local rather than conventional neck dissection. In our case, Tc-99m sestamibi SPECT helped us in identifying the right inferior parathyroid adenoma. 

Once the hypersecreting parathyroid adenoma is identified, surgical excision of the same is the treatment of choice. Prior to surgery, supreme importance is given to stabilize the patient and correct the electrolyte abnormalities. This includes correction of hypercalcemia by adequate hydration, forced calciuresis by loop diuretics, and prevention of further bone resorption by bisphosphonates. In refractory conditions and patients with compromised renal function, hemodialysis can be a quicker way to control hypercalcemia where forced calciuresis is not possible. This is coordinated simultaneously with the management of acute pancreatitis. Studies have shown that adequate rehydration, calciuresis, and bisphosphonate therapy act as a bridge to definitive surgery [14]. Gasparri et al. found that with appropriate initial medical management before surgical excision, mortality rates have fallen to 2.8% among patients with the parathyroid crisis [15]. In our scenario, hypercalcemia was corrected by a combination of intravenous fluids, forced calciuresis, and hemodialysis. Simultaneously, the acute episode of pancreatitis was managed conservatively. Definitive treatment by surgical excision of the parathyroid adenoma was done after stabilization of our patient’s general condition.

The learning points from this case report include: acute pancreatitis should be considered in all patients presenting with acute abdomen; serum calcium levels should be estimated as a part of routine work-up in such patients irrespective of the age at presentation; a contrast-enhanced computed tomography (CECT) of the abdomen should be done in all patients of acute abdomen before subjecting them to laparotomy; in centers without the facility for an emergency CECT, every effort should be made to rule out acute pancreatitis by USG and biochemical evaluation in these patients; in patients with acute pancreatitis, investigations for rare causes should be explored when the cause could not be attributed to common conditions like gallstone disease or alcoholism; combined medical and surgical treatment remains the cornerstone in the management of hyperparathyroidism associated acute pancreatitis. 

## Conclusions

The presence of hypercalcemia in the background of acute pancreatitis brought to light the hidden parathyroid adenoma with PHPT. Similarly, in circumstances where there exists an etiological doubt of acute pancreatitis, infrequent causes must be considered for evaluation. Early diagnosis and prompt treatment of the underlying condition can prevent recurrence and further due complications of pancreatitis.
